# Does the built environment of settlements affect our sentiments? A multi-level and non-linear analysis of Xiamen, China, using social media data

**DOI:** 10.3389/fpubh.2022.1094036

**Published:** 2023-01-06

**Authors:** Chenjing Fan, Zhenyu Gai, Shiqi Li, Yirui Cao, Yueying Gu, Chenxi Jin, Yiyang Zhang, Yanling Ge, Lin Zhou

**Affiliations:** ^1^School of Landscape Architecture, Nanjing Forestry University, Nanjing, China; ^2^School of Public Administration and Policy, Renmin University of China, Beijing, China; ^3^Institute of Industrial Economics of Chinese Academy of Social Sciences, Beijing, China

**Keywords:** neighborhoods, subdistricts, sentiment analysis, natural language processing, social media text data

## Abstract

**Introduction:**

Humans spend most of their time in settlements, and the built environment of settlements may affect the residents' sentiments. Research in this field is interdisciplinary, integrating urban planning and public health. However, it has been limited by the difficulty of quantifying subjective sentiments and the small sample size.

**Methods:**

This study uses 147,613 Weibo text check-ins in Xiamen from 2017 to quantify residents' sentiments in 1,096 neighborhoods in the city. A multilevel regression model and gradient boosting decision tree (GBDT) model are used to investigate the multilevel and nonlinear effects of the built environment of neighborhoods and subdistricts on residents' sentiments.

**Results:**

The results show the following: (1) The multilevel regression model indicates that at the neighborhood level, a high land value, low plot ratio, low population density, and neighborhoods close to water are more likely to improve the residents' sentiments. At the subdistrict level, more green space and commercial land, less industry, higher building density and road density, and a smaller migrant population are more likely to promote positive sentiments. Approximately 19% of the total variance in the sentiments occurred among subdistricts. (2) The proportion of green space and commercial land, and the density of buildings and roads are linearly correlated with residents' sentiments. The land value is a basic need and exhibits a nonlinear correlation with sentiments. The plot ratio, population density, and the proportions of industrial land and the migrant population are advanced needs and are nonlinearly correlated with sentiments.

**Discussion:**

The quantitative analysis of sentiments enables setting a threshold of the influence of the built environment on residents' sentiments in neighborhoods and surrounding areas. Our results provide data support for urban planning and implementing targeted measures to improve the living environment of residents.

## 1. Introduction

As an important comprehensive indicator to measure people's quality of life and mental health, subjective sentiments have attracted attention from various fields. According to the World Health Organization, 322 million people were affected by depression worldwide in 2017 (1). Due to rapid urbanization, the number of people with depression in China has reached 54 million; 73.6% are in a state of psychological sub-health, and 16.1% have psychological problems of varying degrees (2). Although residents' subjective sentiments are largely influenced by individual and family status, such as income, marriage, age, economic status, genetic indicators, and individual subjective indicators, environmental indicators comprise 40–50% of all factors affecting subjective sentiments (3). Studies have shown that people spend an average of 87% of their time indoors and about half of their time in their neighborhoods due to increasing urbanization (4). There is widespread awareness that improvements in the built environment of neighborhoods may improve the residents' sentiments. Research on the relationship between the living environment and psychological factors is a core topic in urban planning and environmental psychology, and the improvement of residents' sentiments is one goal of urban planning. Research on sentiments and living environments encompasses areas as diverse as inequality (5), space deprivation (6), and policy, which are critical to the health of rapidly urbanizing cities in developing countries.

Empirical studies based on questionnaires have shown that land prices (7, 8), location (9, 10), spatial form (11), and the built environment (such as the plot ratio, greening rate, property ownership, transportation organization, and density) (12–14) in neighborhoods may affect residents' sentiments. However, the largest challenge in this field is the large-scale quantification of sentiments. The concept of subjective sentiments has been typically used to evaluate an individual's sentiments (15). However, subjective sentiments are difficult to monitor or quantify in real time. For example, most studies relied on questionnaires, which have limitations, such as limited quantitative measurements, low coverage, recovery, and efficiency, and difficulty in replicating the results. In the past 2 years, few studies have used big data to quantify the sentiments of urban residents (16, 17). However, these data have rarely been used to conduct neighborhood-level research on residents' sentiments. More importantly, as discussed in the next section, objective, large-sample, non-discrete, and reproducible quantitative sentiment measurements may provide a detailed reference for studying multilevel and non-linear relationships between the built environment and sentiments.

This study uses social media text data and natural language processing (NLP) to quantify the residents' sentiments in Xiamen, China. A multilevel regression model is established at the neighborhood and subdistrict levels to investigate the relationship between the built environment and residents' sentiments at different levels. We use the gradient boosting decision tree (GBDT) model to evaluate the non-linear correlation between variables with a significant impact. We attempt to answer the following questions: (1) What is the sentiment difference between residents living in different subdistricts of the city? (2) Which are the built environment indicators affecting individual subjective sentiments at the neighborhood and subdistrict levels? (3) Is the relationship between the built environment indicators and the sentiments non-linear or linear? This study uses objective social media text data to quantify long-term sentiments instead of short-term happiness (18) to provide a reference for interdisciplinary research on urban planning and public mental health. In addition, determining whether the relationship between the sentiments and the built environment at multiple levels is linear or non-linear is critical for optimizing the built environment of neighborhoods to improve residents' sentiments.

The rest of this article is organized as follows. Section 2 is a literature review of the quantification of sentiments, the built environment at multiple levels, and non-linear studies of sentiments to identify current research problems. Section 3 introduces the data, sentiment quantification methods, variables of the built environment, and multilevel and non-linear regression modeling methods. Sections 4 and 5 present the results and discussions. The final section summarizes the paper and discusses policy implications.

## 2. Literature review

### 2.1. Research on the quantitative analysis of residents' sentiments

Questionnaires are commonly used in sentiment studies and social research (19). However, this method may not be objective and may not reflect the psychological state of the subjects. The results of psychological state studies are influenced by the subjective feelings of the research participants and by the questionnaire design. Uncertain and confounding indicators may exist, such as the same questions applicable to different environmental conditions and inappropriate measurement methods. Moreover, discrete variables are typically used in questionnaires (20). Thus, the results need to be reclassified and scored to conform to a normal distribution and meet the requirements of statistical inference. A reproducible quantitative method can avoid some of the shortcomings of traditional surveys.

A limited number of studies have used social media data for sentiment analysis to determine the objective sentiments of residents in cities. Social media capture thousands of interactions between individuals and large groups over a long period. Semantic analysis has been used to analyze large samples of highly objective and spatially and temporally resolved data to study the sentiments and wellbeing of individuals and societies. These data can be used to assess mental health and public sentiments (21, 22). One advantage of using social media data rather than questionnaires and interviews is the large sample size for analyzing sentiments (23). Text analysis and geographical analysis have been used to process social media data to obtain non-discrete and reproducible quantitative sentiment data with high spatial and temporal resolution (24). In general, the use of social media data to quantify sentiments is a widely used and accepted method in the academic community.

### 2.2. Complex relationship between the built environment and residents' sentiments

#### 2.2.1. Multilevel analysis of the impact of the built environment on residents' sentiments

Studies on the influence of the built environment on residents' sentiments have been conducted primarily at two levels: the neighborhood and the surrounding environment (25). Baker and Steemers (26) stated that “In Britain, we spend, on average, as much as 90% of our time inside buildings, 70% of it in our own homes” (26). Therefore, the built environment of a neighborhood probably has the largest influence on residents' sentiments. Most studies are in agreement. For example, it has been shown that unsafe, inadequate facilities and poorly designed landscapes can significantly reduce residents' sentiments, potentially leading to psychological stress and mental problems. In contrast, environments with well-designed facilities, beautiful landscapes, low noise, and more daylight are more likely to evoke positive sentiments (27, 28). However, some disagreement exists on the effect of some indicators, such as the impact of building density indicators on sentiments. Most studies found that a higher building density is more unpleasant and results in negative sentiments (29, 30). However, a study conducted in Oslo reported that high building density might promote social relationships to improve residents' sentiments, provided that the environment is safe and not noisy (31).

Urban planning considers spaces outside neighborhoods (e.g., subdistricts, districts, towns) to enrich residents' daily activities (32). Research has focused on five aspects: land use, spatial form, development intensity, property ownership, and transportation organization (Table 1). (1) Early studies focusing on land use have consistently shown that neighborhoods far away from industrial areas have better public health. Safety and welfare can promote a clean environment and improve the quality of life (33, 34). A large amount of urban green space in neighborhoods can provide good air quality and landscape conditions to enhance sentiments (35–37). In contrast, disagreement has developed over the impact of commercial land use on residents' sentiments. Some studies have found that areas of commercial land around neighborhoods can promote travel, reduce the dependence on cars, and lower residents' travel time and costs (38). However, the proximity of commercial land to residential sites results in more litter, high traffic noise, and low visual quality, potentially evoking negative sentiments (37). (2) Studies on the spatial form found that a large proportion of mixed land use reduced the average walking distance of residents from their homes to sites of interest and increased social interaction, improving the residents' sentiments (39). However, some empirical studies did not produce consistent results. Foord (40) observed that mixed land use improved the convenience and diversity of amenities for residents to meet their lifestyle needs. Cao (41) found that mixed land use in the Twin Cities, MN, provided more amenities but also resulted in more noise, traffic congestion, and possibly stranger danger, resulting in positive and negative effects on the residents' sentiments. However, the overall impact was statistically insignificant. (3) Higher development intensity results in higher population density and diverse impacts on the residents' sentiments. Some studies reported that a higher population density caused overcrowding, unemployment, poverty, and mental stress (31, 42, 43). Other studies suggest that a higher population density may improve residents' sentiments by enabling them to walk through their neighborhoods (23, 44, 45). (4) Some studies on property ownership reported that the migrant population caused a sense of insecurity and instability, increasing the mental stress of residents. When the proportion of the migrant population reached a specific size, the formation of group identity caused a stabilization of the sentiments (46, 47). (5) Many studies have examined the impact of road design on residents' sentiments. Some found that a higher road density provided increased connectivity between neighborhoods and significantly reduced congestion, improving residents' sentiments. It has also been argued that a high road density in neighborhoods can reduce the quality of life in a subdistrict due to landscape fragmentation. Too many road crossings can reduce access efficiency and make residents' travel experiences less enjoyable (48, 49). It has also been found that residents' sentiments are considerably influenced by traffic efficiency (e.g., rush hour, and traffic lights) (50) and that transportation organization and sentiments may not be correlated.

**Table 1:** Studies of the relationship between the built environment and residents' sentiments.

**Indicators**	**Positive correlation**	**Negative correlation**	**Non-significance**
**Built environment of the neighborhood**			
Land value	(8), (7), (20)^*^		
Plot ratio		(25), (10)	
Landscape	(27), (28), (20)^*^, (10)		
**Land use**			
Green space	(33), (34), (10)		
Industrial land		(51)	
Commercial land	(38)	(37)	
**Spatial form**			
Mixed land use	(40)	(10)	(41), (52)
Accessibility of facilities	(46), (47), (53)^*^		
**Development intensity**			
Building density	(31)	(43), (42)	
Population density	(54), (55)	(30), (53)^*^	
**Property ownership**			
Proportion of migrant population		(56), (57)	
**Transportation organization**			
Road density	(49), (58)	(59)	(50), (60)
Road intersections		(48), (61)	

* Indicates studies with non-linear correlations.

Early empirical studies primarily used simple linear regression models to explore the impact of the built environment on residents' sentiments. Subsequently, more complex regression models, such as multiple linear regression and structural equations, were used (62, 63). Although theoretical and empirical evidence suggests that the impact of the built environment on sentiments is multilayered, most studies focused on a single level and individuals.

#### 2.2.2. Studies on the non-linear correlation between the built environment and sentiments

Existing studies show that the built environment has non-linear relationships with overall sentiments (64). Referring to Maslow's theory, residents' needs regarding the environment can be divided into three categories: basic needs, intermediate needs, and advanced needs (65). Negative sentiments occur when basic needs are not met and vice versa. Positive sentiments occur when advanced needs are met, but negative sentiments do not occur when they are not met. Negative sentiments occur when intermediate needs are not met and vice versa (20, 66). Studies on residential environments found a non-linear relationship between negative sentiments and basic needs such as street lighting, residential safety, absence of noise, and nearby facilities (53, 67). A non-linear relationship was also observed between sentiments and advanced needs, such as diverse architectural styles, outstanding education, and good streetscape design (68, 69). However, questionnaire methods used in most sentiment analysis studies provide mostly discrete data, and there may be errors in analyzing non-linear relationships. Using an ordinal scale to classify the sentiments of residents is subjective and does not provide a trend, making it difficult to determine whether the variance in the data is the result of random errors or curve fitting when assessing non-linear relationships.

Although theoretical and empirical studies in public health and planning indicate a multilevel and non-linear correlation between the spatial environment and residents' short-term satisfaction, most multi-level analyses have focused on the individual and the environment. In contrast, the impact of multi-level differences in the spatial environment on residents' long-term sentiments has been based on theoretical approaches, and few empirical studies have been conducted. However, public health studies focused more on long-term sentiments than short-term satisfaction (18). Researchers started to use social media data to analyze the long-term sentiments of people ~2 years after the development of NLP techniques (70–72). These studies found complex relationships between people's sentiments and the built environment in boroughs with different region, no quantitative analysis was conducted. Furthermore, most studies used discrete data from questionnaires, which are highly subjective and contingent, making it difficult to replicate the results and assess the non-linear relationship between sentiments and the environment. Assessing the variable and non-linear relationship between the different elements of the built environment and the multi-level needs of residents requires more accurate and comprehensive data.

## 3. Data and methods

**Figure 1:**
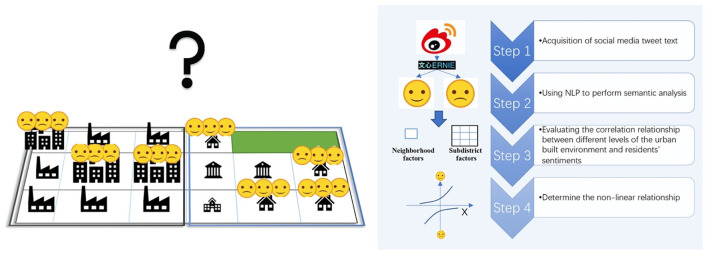
Technical route.

We use tweet text data from social media platforms and the sentiment knowledge enhanced pretraining (SKEP) algorithm to score the sentiments. Multilevel regression analysis and non-linear correlation analysis are used to assess the relationship between the built environment and residents' sentiments. The approach uses 4 steps: (1) Acquisition of social media tweet texts and data cleaning. (2) Using NLP to perform semantic analysis of the social media tweets. (3) Establishing a multilevel regression model to evaluate the correlation relationship between different levels of the urban built environment and residents' sentiments. (4) Using the GBDT model to determine the non-linear relationship between different indicators of the urban built environment and residents' sentiments (Figure 1).

### 3.1. Study area

**Figure 2:**
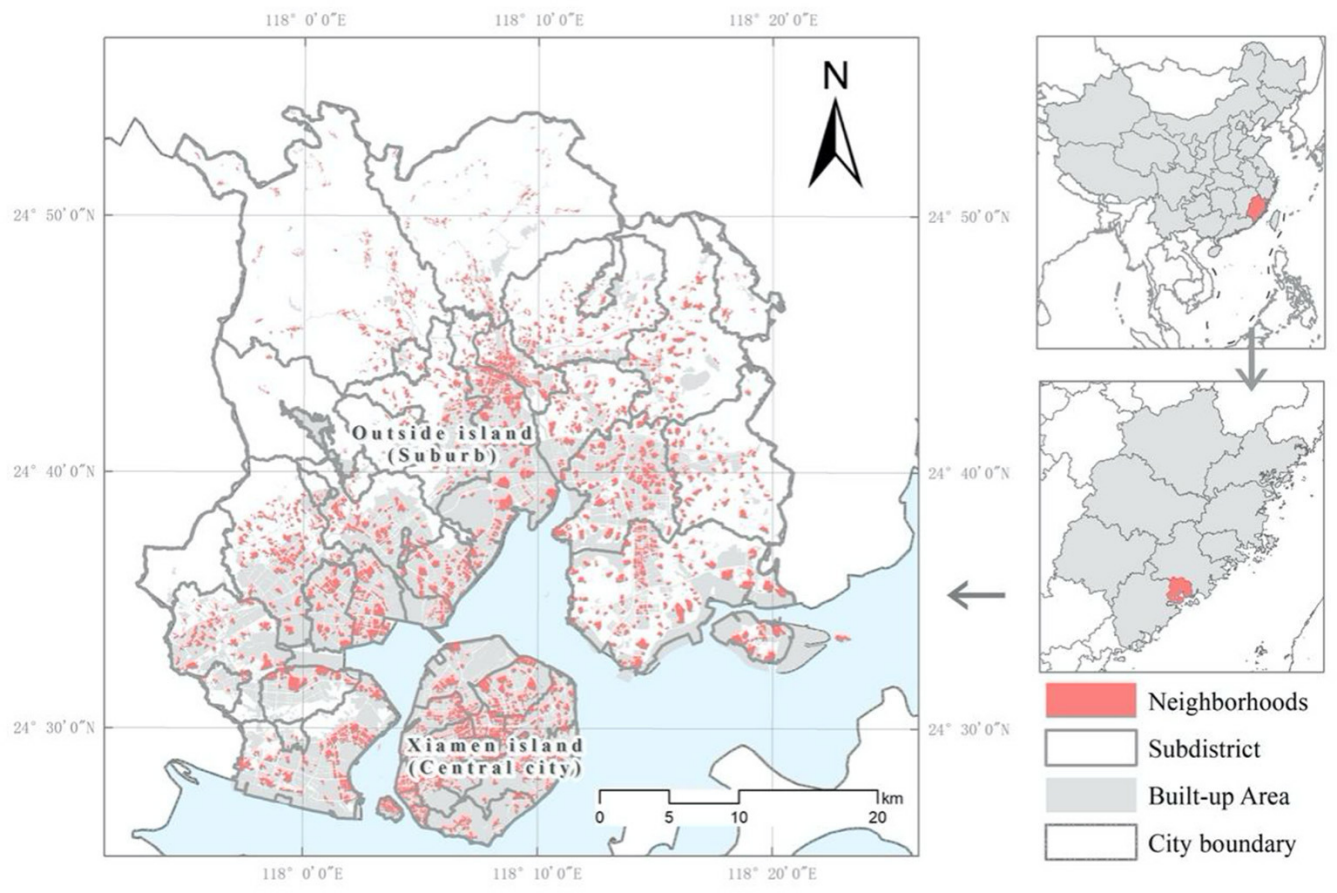
Study area.

The study area is Xiamen, China. Xiamen is located in East China in the southeast of Fujian Province; it has 50 subdistricts. The city covers an area of 1,699.39 square kilometers, and the permanent population of Xiamen was 4.01 million in 2017. Xiamen has repeatedly ranked first in China's economic life survey as the happiest city in China. It has an excellent urban environment, a comfortable climate, and is a safe city with high urban development and numerous social and cultural activities (73). In addition, Xiamen's urban development ranks high in China, attracting a large migrant population for work. The complex population structure has also contributed to the formation of many urban villages, which are called *Chengzhongcun* in China (74). These are high-density villages surrounded by urban communities that have poor living conditions and are located in areas with high land prices. In addition, the old city of Xiamen is limited by the terrain, and the building density and housing prices are much higher than those of the new city. Therefore, the built environment of Xiamen is highly unbalanced and complex, making it ideal for this type of research. Many studies on the residents' sentiments and lives focused on tourism and housing have been conducted in Xiamen (70, 75, 76). We used the 2018 land-use map of Xiamen City to select the high-grade and medium-grade residential land (Code R1 and R2) and urban villages (Code R3) for this research (Figure 2).

### 3.2. Data sources

#### 3.2.1. Data of residents' sentiments obtained from social media tweets

Social media tweet data include the social media user's geographic information (latitude and longitude coordinates), time, and text. Since check-in events are based on people's conscious behavior, people only post at a location if they stay for a relatively long time and think they have information worth recording. Thus, these tweets reflect the user's psychological state. The amount of data is larger than that obtained from questionnaires. Unlike mobile phone data and night lights, social media check-in data contain information on human sentiments; thus, they are more suitable for studying the state of residents (77, 78).

We used Sina Weibo check-in data. Sina Weibo is a widely used social networking platform in China. Users are encouraged to check in frequently, recording their daily activity patterns and behaviors. The data used in this article was obtained by crawling the annual Weibo tweet data using the Sina Weibo Application Programming Interface (API) in 2017, including public data, such as Weibo tweets, generation time, user ID, and location. The data were filtered to remove repetitive, garbled, and other meaningless text or symbols in the text, such as URLs, Html, tags, curly single or double quotes, email addresses, and non-ASCII characters (8, 79). The Weibo data with a neighborhood was extracted, and a total of 146,147 tweets were obtained. There were 1,096 neighborhoods with more than 10 tweets, exceeding the amount of data obtained from a questionnaire.

The quantification of the residents' sentiments was performed using deep learning classifiers. They can identify emojis and text in social media tweets, analyze subjective texts with sentiment overtones, and score the sentiments (70). We used the open access model SKEP based on ERNIE 3.0 to analyze the sentiments (80, 81). First, we used the pre-trained model dataset to build a learning platform based on sentiment knowledge. Then, we manually labeled 50,000 Weibo texts with sentiments. Machine learning based on pre-trained samples teaches computers how to quantify sentiments in tweets (82). Unlike scoring methods, such as sentiment dictionaries and cloud sentiment analysis (8, 83), the SKEP method is similar to human subjective thinking, and the method is reproducible. The sentiment scores of the tweet text have a range of [0, 1]. The closer the score is to 1, the more positive the sentiments are, and vice versa. The sentiment analysis preprocessing model was shared in Figshare (https://doi.org/10.6084/m9.figshare.21524391). A comparison of the publicly available Chinese corpus (https://console.bce.baidu.com/ai/?_=1667982697826&fromai=1#/ai/nlp/sentiment/dict/list) annotated by Baidu Intelligent Cloud Sentiment analysis showed that the classification accuracy of the SKEP model was 95.8%. The precision and recall for the positive sentiments were 95.7 and 96.4%, and those for the negative sentiments were 96 and 95.1%, respectively, demonstrating the reliability of the sentiment classification results are reliable.

#### 3.2.2. Built environment data

We obtained statistical data for the neighborhood and subdistricts (Table 1). In China, a neighborhood is the smallest residential unit for urban planning and management. It is constructed by a developer or the village collective, and the boundaries of the neighborhood determine in urban planning depend on the land granted. A subdistrict consists of several neighborhoods and is an administrative unit in China. It is subject to the urban planning goals of the government.

The neighborhood data was obtained from the website of *Fangtianxia*, a Chinese real estate market website (https://www.fang.com/). Typically, Internet real estate data are collected by designated staff and uploaded to the Internet by relevant institutions. The data include neighborhood construction information, such as neighborhood location, boundary, land value, number of households, building density, plot ratio, and greening rate. We extracted the neighborhood boundaries from the current map of the urban master plan and calculated the information on living facilities, population, public facilities, and other information within 1 km of the neighborhood.

Urban dwellers spend most of their time in neighborhoods that provide the most important functions of daily life. Multiple indicators affect sentiments (Table 1). For example, the housing area, building density, plot ratio, and greening rate may affect the living experience of residents. The number of households, the proportion of the migrant population, and population density affect residents' social activities. Property fees and land values affect the living expenses of residents. Cultural, public, and transportation facilities affect the living experience of residents. We used two types of indicator statistics; one is a numerical variable for the number of neighborhoods, population structure, and land value. The other is a categorical variable, e.g., for cultural facilities and proximity to water.

The subdistrict characteristics were extracted from the 2018 land-use map of Xiamen City, including the subdistrict area, name, and region. We derived statistics for the subdistrict, including land use, spatial form, development intensity, property ownership, and transportation. The average of the sentiments for each neighborhood was used as the dependent variable.

Since a subdistrict is used by urban residents for jurisdiction and communication activities, the spatial elements have the largest impact on the residents' sentiments. For example, different land-use types of the subdistrict can provide different functions. The public service facilities may affect the residents' accessibility, and the building density may reflect the development level of the subdistrict. The proportion of the migrant population in the subdistrict may affect the interpersonal experiences of residents, and road density may affect traffic quality.

The indicators and their calculation are listed in Table 2; *X*_*n*_ is a neighborhood-level indicator, and *S*_*n*_ is a subdistrict-level indicator.

**Table 2:** Research variables and their calculation methods.

**Level**	**Type**	**Indicator**	**Code**	**Unit**	**Calculation method**
Dependent variable		Sentiment score	*Y*	-	Quantitative analysis of social media tweet data, details in 3.2.
Neighborhood level	Land value	Land value	*X*_1_	10,000 RMB yuan	Average housing prices in public neighborhoods obtained from real estate websites (https://www.fang.com/). The price of houses that cannot be sold, such as urban villages, are estimated using spatial interpolation.
	Neighborhood built environment	Plot ratio	*X*_2_	-	Total neighborhood building area/neighborhood land area. The data was obtained from real estate websites (https://www.fang.com/).
		Population density	*X*_3_	Number of people per hectare	Neighborhood population/neighborhood land area. The data was obtained from real estate websites (https://www.fang.com/).
		Proximity to water	*X*_4_	-	0 = Close to the water. 1 = Not close to the water.
Subdistrict level	Land use	Proportion of green space	*S*_1_	%	Total area of green space/total area of the subdistrict. The data was obtained from land-use maps.
		Proportion of industrial land	*S*_2_	%	Total area of industrial land/total area of the subdistrict. The data was obtained from land-use maps.
		Proportion of commercial land	*S*_3_	%	Total area of commercial land / total area of the subdistrict. The data is derived from land-use maps.
	Spatial form	Mixed land use	*S*_4_	-	We used the information entropy Shannon-Wiener Index to calculate it: *H* = −∑(*P*_*i*_)(ln *P*_*i*_), where *P*_*i*_ is the proportion of land use derived from land-use maps.
		Number of public service facilities	*S*_5_	Number	The number of public services in the subdistrict derived from the facilities in the current map.
	Development intensity	Building density	*S*_6_	%	Subdistrict floor area/total subdistrict area. Building density is derived from housing boundaries calculated by the Housing and Urban-Rural Development Bureau.
	Property ownership	Proportion of migrant population	*S*_7_	%	Number of migrant population/total subdistrict population. The data was obtained from the 2017 Population Census.
	Transportation organization	Road density	*S*_8_	m/km^2^	Total subdistrict road length/total subdistrict area. The data was obtained from the Traffic Bureau's road status map.

- Indicates that the indicator is dimensionless.

### 3.3. Statistical analysis of the correlation between residents' sentiments and built environment indicators

#### 3.3.1. Multilevel regression model

The sentiments of urban dwellers may have a multilevel relationship with the built environment. Some scholars have conducted hierarchical studies on the built environment at different administrative levels and under different development conditions to determine the impact of the built environment factors on residents' sentiments at multiple levels (20, 84). Our sample data had a hierarchical structure, with low-level neighborhood data nested within high-level subdistrict data. The objective of this study is to determine the effect of the built environment on the population's happiness. Thus, we focus more on public health (18) than individual attributes or satification (85). Therefore, a multilevel regression model was used to analyze the differences between subdistricts and neighborhoods. Multi-level regression models can also explain the relationship of variables at different levels with the dependent variable. As a result, we used neighborhood-level indicators and subdistrict-level indicators to construct a two-level regression model.

The first step is to analyze the data hierarchy using an empty model with no explanatory variables. In this case, there are two levels; *i* is the neighborhood, and *j* is the subdistrict. *Y*_*ij*_ represents the observed variable of neighborhood *i* in subdistrict *j*. The model is defined as:


(1) $$\begin{array}{l} Y_{ij}=\beta_{0j}+\varepsilon_{ij}. \end{array}$$

The change in the intercept between neighborhoods can be expressed as:


(2) $$\begin{array}{l} \beta_{i0}=\gamma_{00}+u_{i0}. \end{array}$$

The empty model is defined as:


(3) $$\begin{array}{l} Y_{ij}=\gamma_{00}+u_{i0}+\varepsilon_{ij}. \end{array}$$

where γ_00_ is the mean intercept, β_*i*0_ is the neighborhood-level intercept, *u*_*i*0_ is the random effect of the neighborhood-level intercept, and ε_*ij*_ represents the estimated neighborhood-level difference in the built environment.

The inter-group correlation coefficient (ICC) can be calculated using the empty model. It is defined as the ratio of the variance between groups to the total variance:


(4) $$\begin{array}{l} ICC=\frac{\sigma_{0j}^{2}}{\left(\sigma_{0j}^{2}+\sigma^{2}\right)}. \end{array}$$

The empty model [Equation (3)] can estimate the variation across all subdistricts. $\sigma_{0j}^{2}$ is the neighborhood-level variance, and σ^2^ is the subdistrict-level variance. The significance of ${\hat{\sigma }}_{0j}$ and the size of the *ICC* determines whether the sentiment difference is significantly affected by the subdistrict and whether a multi-level model is required.

If there is a difference, a subdistrict-level variable is added to Equation (3) to create Equation (5). Five models are constructed: land use, spatial form, development intensity, property ownership, and transportation organization. The significant built environment variables at the neighborhood level are retained, and the subdistrict-level variables are added.


(5) $$\begin{array}{l} \beta_{i0}=\gamma_{00}+\gamma_{i0}S_{j}{+u}_{i0}. \end{array}$$

(6) $$\begin{array}{l} Y_{ij}=\beta_{i0}+\gamma_{0j}X_{i}+\varepsilon_{ij}. \end{array}$$

Equations (5) and (6) are combined to obtain the final model:


(7) $$\begin{array}{l} Y_{ij}=\gamma_{00}+\gamma_{0j}X_{i}+\gamma_{i0}S_{j}+u_{i0}+\varepsilon_{ij}. \end{array}$$

where *X*_*j*_ denotes the neighborhood variables, and *S*_*i*_ denotes the subdistrict-level variables that remain in the final model only if they are significant. γ_00_+γ_0*j*_*X*_*i*_+γ_*i*0_*S*_*j*_ is the fixed effect, γ_0*j*_ is the main effect of explanatory variable *X*_*i*_ at the neighborhood level, γ_*i*0_ is the main effect of the explanatory variable *S*_*j*_ at level 2, and *u*_*i*0_+ε_*ij*_ is the random effects.

An empty model was constructed to determine the difference in people's sentiments between neighborhoods and subdistricts. We established a single-level model with five indicators at the neighborhood level. We then added variables at the subdistrict level (land use, spatial form, development intensity, property ownership, and transportation organization) to establish different models. Several indicators were removed to prevent multicollinearity (86, 87).

#### 3.3.2. Non-linear GBDT regression model

The sentiments of city dwellers may have a non-linear relationship with the built environment. We used the GBDT method to describe the non-linear relationship between sentiments and neighborhood spatial features. The GBDT uses decision trees and gradient boosting regression trees. The gradient boosting is based on the residuals of the previous tree (53). We used the mean of the response variable to predict and calculate the residuals and determine the difference between the observed and predicted values. Then, a tree was added to predict the residuals. The new predicted value of the response was the sum of the predicted values from the previous step. The predicted residuals were multiplied by the learning rate (a number from 0 to 1), and the new residuals were obtained by subtracting the new predicted values from the observed values. We repeated the second step until the addition of a tree did not improve the prediction result or the maximum number of trees was reached. The GBDT method uses additive regression models by sequentially fitting a simple parametric function to the current residuals using least squares at each iteration (88). We selected index *x*_*i*_ with a significant correlation in the multi-level regression model.

First, the optimal constant model (Equation 8) was initialized to minimize the loss function *L*(*Y*_*ij*_, γ).


(8) $$\begin{array}{l} f_{0}\left(x\right)=\arg\underset{\gamma }{\min}\underset{N}{\sum }L\left(Y_{ij},\gamma \right). \end{array}$$

In the second step, each iteration of m had four sub-steps (a - d). First, we calculated the negative gradient using Equation (9). Next, we fit a regression tree to the target. The third sub-step was to calculate the gradient descent step size based on different tree expansions using Equation (10). In the last sub-step, Equation 11, the model was updated based on the results of Equation (10).

For m = 1 to M:

(a) For i = 1, 2, …, N, calculate the pseudo-residuals


(9) $$\begin{array}{l} r_{im}=-{\left[\frac{\partial L\left(Y_{ij},f\left(x_{i}\right)\right)}{\partial f\left(x_{i}\right)}\right]}_{f=f_{m-1}}. \end{array}$$

(b) Fit the target edge of the regression tree and determine the terminal area *R*_*jm*_, *j* = 1, 2, …, *m*.

(c) For j = 1, 2, …, *m*, compute


(10) $$\begin{array}{l} \gamma_{jm}=\arg\underset{y}{\min}\underset{x\in R_{jm}}{\sum }L\left(Y_{ij},f_{m-1}\left(x_{i}\right)+\gamma \right). \end{array}$$

(d) Update


(11) $$\begin{array}{l} f_{m}\left(x_{i}\right)=f_{m-1}\left(x_{i}\right)+\overset{J_{m}}{\underset{j=1}{\sum }}\gamma_{jm}I\left(x_{i}\in R_{jm}\right). \end{array}$$

The third step is to generate the final model as follows:


(12) $$\begin{array}{l} \hat{f}\left(x_{i}\right)=f_{M}\left(x_{i}\right). \end{array}$$

where *Y*_*ij*_ is the sentiment mean, γ is the step size, *r*_*im*_ is the pseudo-residual, *R*_*jm*_ is the terminal region, *M* is the number of iterations, *N* is the number of eigenvalues, and *J* is the size of each constituent tree.

**Figure 3:**
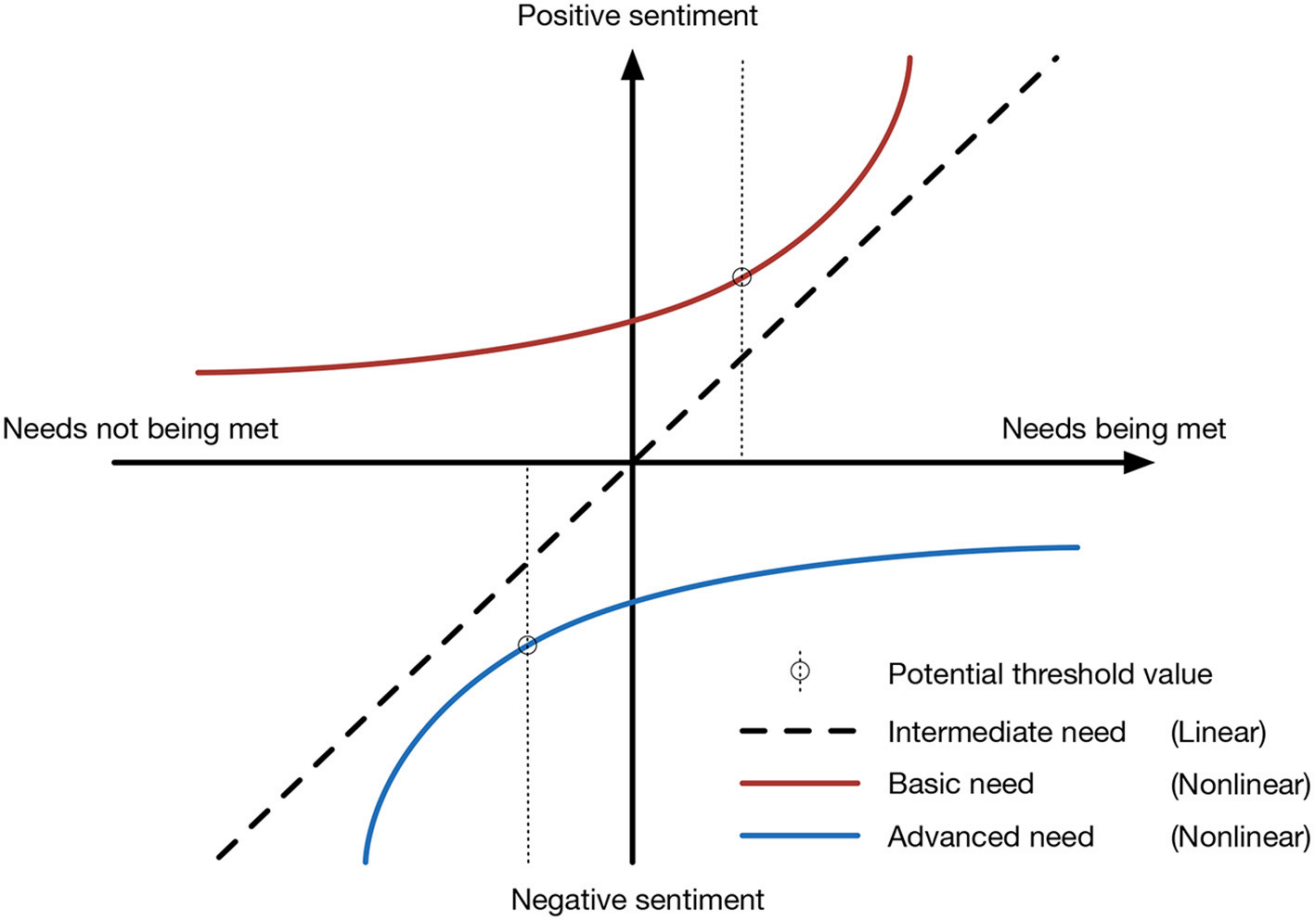
Three correlations between residents' needs and sentiments.

The regression curve was plotted according to the model fitting results, and the influence of different indicators on the sentiments was assessed. Positive sentiments occur when an intermediate need is met and vice versa. According to the three-factor theory, positive sentiments also occur when advanced needs are met, but negative sentiments do not occur when they are not met (89). The thresholds in the non-linear relationships were analyzed (Figure 3).

## 4. Results

### 4.1. Results of variable preprocessing

**Figure 4:**
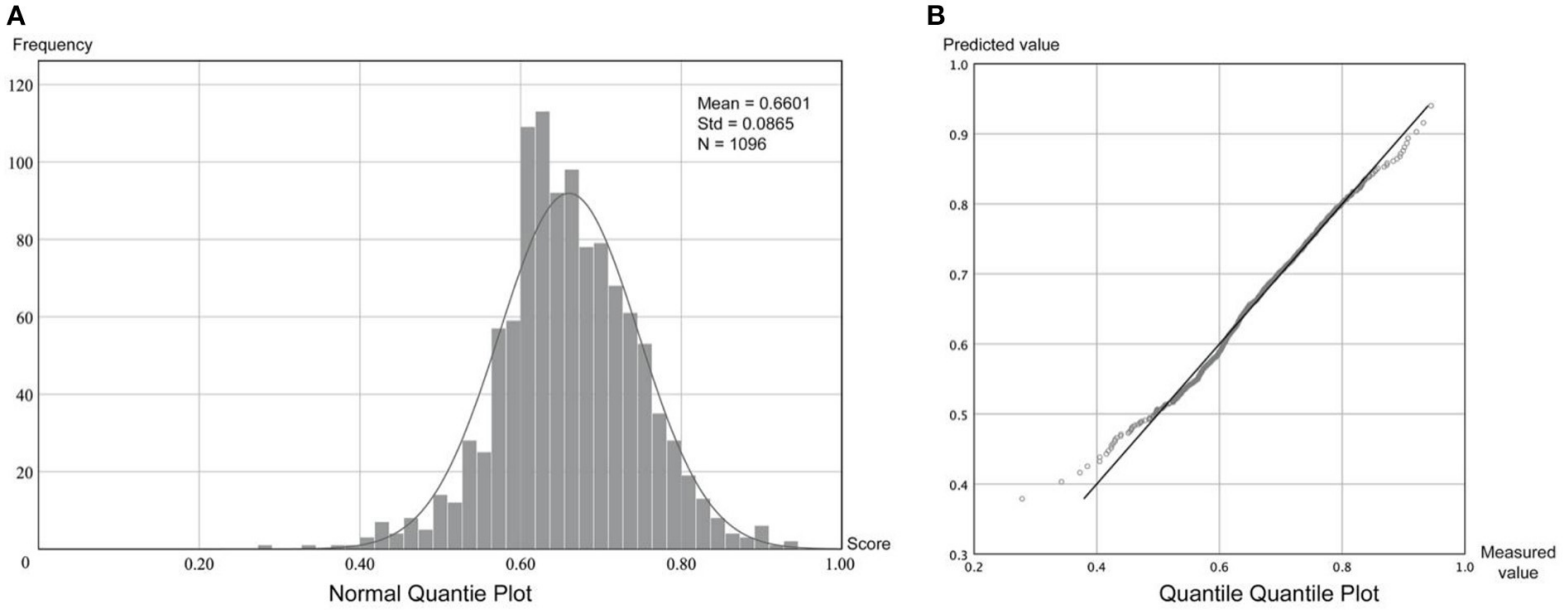
Frequency distribution and normal distribution of residents' sentiments. **(A)** Normal quantile plot. **(B)** Quantile quantile plot.

Table 3 list the descriptive statistics of the mean sentiments and variables at different levels. Figure 4 illustrates the high accuracy of the tweet data classification and the distribution of the non-discrete sentiment data. It was verified that the mean sentiments of the neighborhood followed a normal distribution, and the sample size was sufficiently large to reflect the sentiments of the neighborhood residents.

**Table 3:** Descriptive statistics of research variables.

**Code**	**Indicator**	**Number**	**Min**	**Max**	**Mean**
*Y*	Average sentiment score	1,090	0.28	0.94	0.66
*X*_1_	Land value	1,090	0.70	14.20	4.57
*X*_2_	Plot ratio	1,090	0.00	9.20	1.88
*X*_3_	Population density	1,090	0.00	0.05	0.02
*X*_4_	Proximity to water	1,090	0	1	0.28
*S*_1_	Proportion of green space	50	0.01	52.04	18.01
*S*_2_	Proportion of industrial land	50	0.00	27.48	6.51
*S*_3_	Proportion of commercial land	50	0.00	14.53	5.92
*S*_4_	Mixed land use	50	0.16	3.54	2.43
*S*_5_	Number of public service facilities	50	27	211	102
*S*_6_	Building density	50	0.36	30.16	13.19
*S*_7_	Proportion of migrant population	50	7.40	84.47	45.87
*S*_8_	Road density	50	936.64	27,989.16	13,000.30

**Figure 5:**
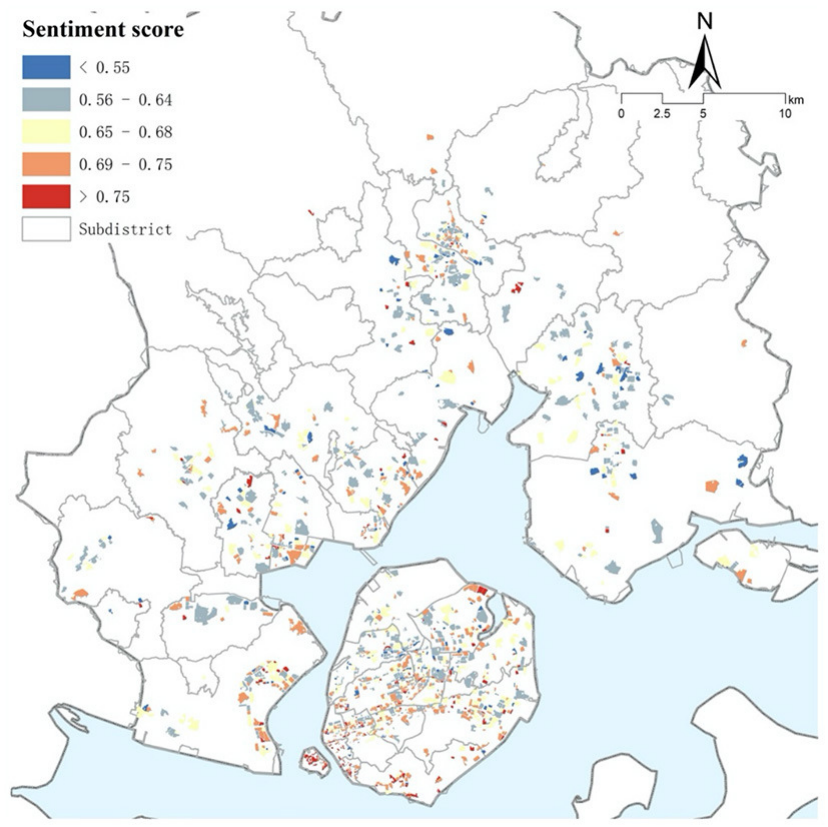
Neighborhood sentiment scores in Xiamen City.

The average sentiment in Xiamen is 0.66, representing an average of 66 positive sentiments per 100 Weibo texts. The average sentiment on Xiamen Island is 0.67, and the average value outside of the island is 0.63, indicating a higher sentiment level on the island than outside the island. Figure 5 depicts the difference in the sentiments for different subdistricts. Therefore, it is necessary to perform multilevel regression.

### 4.2. Multilevel regression results

The multi-level regression results in Table 4 show that in the empty model, the intergroup variance of the two-level regression model is 0.1986, the within-group variance is 0.8270, the ICC is 0.1937, and the *p* < 0.001, indicating that ~19% of the total variance in the sentiments occurred among subdistricts. The variance inflation factor (VIF) of model 1 is <1.5, indicating no multicollinearity between the indicators. At the neighborhood level, the land value and proximity to water are significantly positively correlated with the sentiments. The plot ratio and population density are significantly negatively correlated with the sentiments. At the subdistrict level, the proportion of green space, the proportion of commercial land, building density, and road density are significantly positively correlated with the sentiments. The proportion of industrial land and the proportion of the migrant population are significantly negatively correlated with the sentiments. Mixed land use and the number of public service facilities were not significantly correlated with the sentiments.

**Table 4:** Two-level regression results for neighborhoods and subdistricts.

**Level**	**Dimension**	**Indicator**	**Empty model**	**Model 1**	**Model 2**	**Model 3**	**Model 4**	**Model 5**	**Model 6**	**Model 7**
Level 1:	Land value	Land value		0.290^***^	0.173^***^	0.143^***^	0.299^***^	0.243^***^	0.221^***^	0.256^***^
Neighborhood	Neighborhood built environment	Plot ratio		−0.097^***^	−0.073^*^	−0.078^**^	−0.093^**^	−0.096^***^	−0.075^*^	−0.097^***^
		Population density		−0.085^*^	−0.092^**^	−0.143^***^	−0.082^*^	−0.135^***^	−0.071^*^	−0.132^**^
		Proximity to water		0.152^*^	0.150^*^	—	0.150^*^	0.144^*^	0.140^*^	0.147^*^
Level 2:	Land use	Proportion of green space			0.116^***^					
Subdistrict		Proportion of industrial land			−0.127^***^					
		Proportion of commercial land				0.262^***^				
	Spatial form	Mixed land use					—			
		Number of public service facilities					—			
	Development intensity	Building density						0.106^*^		
	Property ownership	Proportion of migrant population							−0.170^***^	
	Transportation organization	Road density								0.092^*^
		ICC	0.194							
		AIC	2,959.54	3,035.07	3,008.43	2,997.26	3,043.21	3,034.57	3,010.45	3,035.28

— Indicates no significant effect.

*** Represents significant at the 0.001 level,

** represents significant at the 0.01 level, and

* represents significant at the 0.05 level.

### 4.3. Non-linear regression results obtained from GBDT model

According to the results of the multilevel regression model, we selected indicators with significant correlations and used them in the GBDT model to assess the non-linear relationship between different indicators on residents' sentiments.

**Figure 6:**
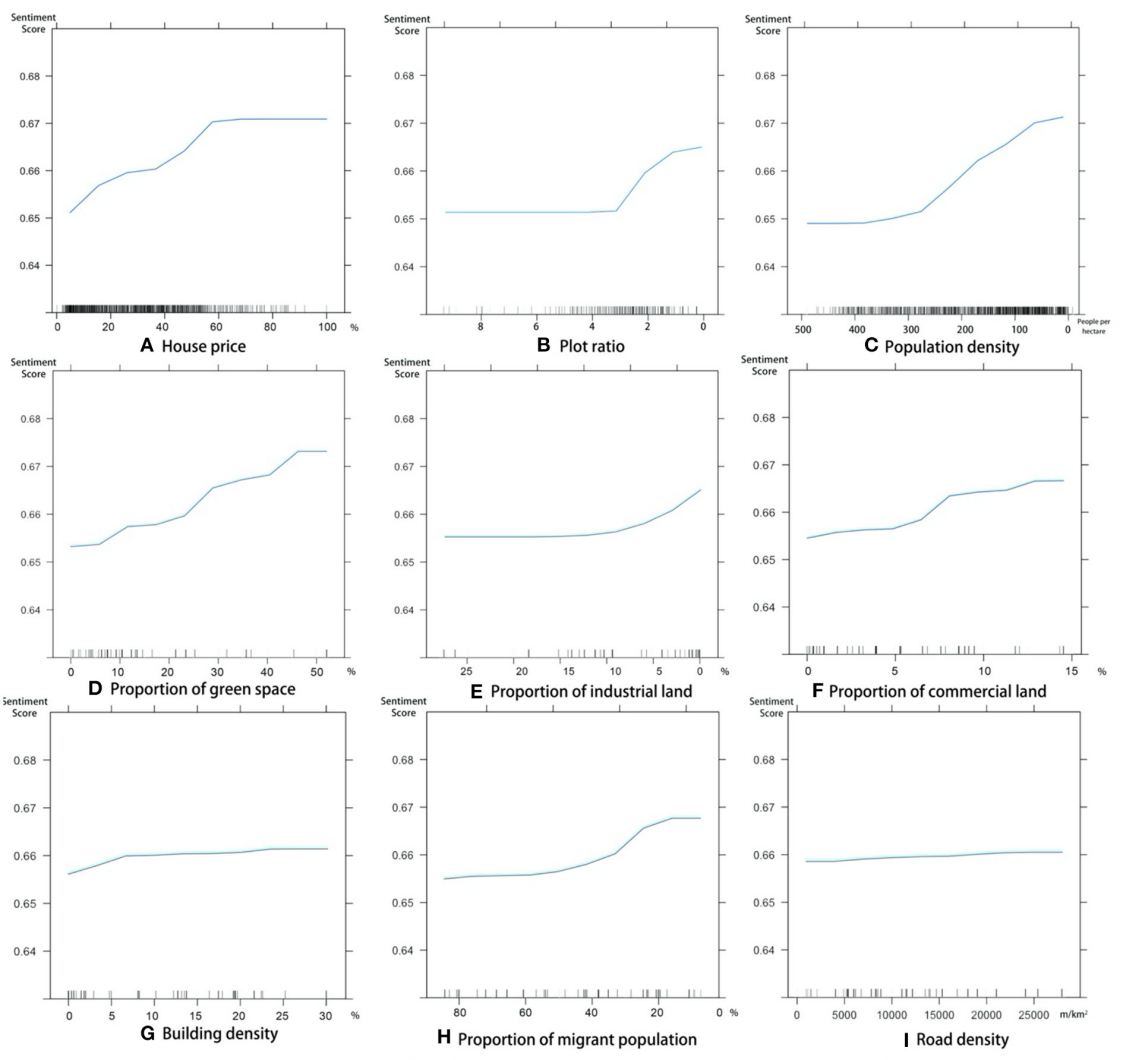
Non-linear correlation between sentiments and different independent variables. [**(A)** land value (percentile); **(B)** plot ratio*; **(C)** population density*; **(D)** proportion of green space; **(E)** proportion of industrial land*; **(F)** proportion of commercial land; **(G)** building density; **(H)** proportion of the migrant population*; **(I)** road density]. *The variable is negatively correlated with sentiments; therefore, we plotted the growth of the built environment in the positive direction of the horizontal axis.

We set the parameters in the GBDT model by focusing on the key parameters, such as the number of trees and the shrinkage, following Fan et al. (89). We used the following GBDT model parameters: the number of trees was 10,000, the shrinkage was 0.0001, and the minimum number of observations in the terminal nodes of the trees was 20. The results are plotted in Figure 6. Since there were positive and negative correlations between different indicators, we plotted the growth of the built environment in the positive direction of the horizontal axis. The larger the indicator value, the more influence it has. We observed different degrees of non-linear correlations between the neighborhood and subdistrict variables that significantly affected the sentiments (Figure 6, Table 5). The land value had a greater impact on sentiments when the indicator value was low, and it was difficult to meet residents' needs. This is referred to as a basic need in the non-linear relationship. Population density, the proportion of industrial land, building density, and the proportion of the migrant population had a larger influence on sentiments when the indicator values were high, i.e., an advanced need in a non-linear relationship. A linear relationship was observed between the sentiments and the proportion of green space and the proportion of commercial land. Table 5 summarizes the non-linear characteristics and thresholds.

**Table 5:** Summary of relationships.

**Level**	**Code**	**Indicator**	**Type**	**Threshold**
Neighborhood	*X*_1_	Land value	Basic need	60%
	*X*_2_	Plot ratio	Advanced need	3
	*X*_3_	Population density	Advanced need	300
Subdistrict	*S*_1_	Proportion of green space	Intermediate need	^*^
	*S*_2_	Proportion of industrial land	Advanced need	10%
	*S*_3_	Proportion of commercial land	Intermediate need	^*^
	*S*_6_	Building density	Intermediate need	^*^
	*S*_7_	Proportion of migrant population	Advanced need	40%
	*S*_8_	Road density	Intermediate need	^*^

* Indicates that the indicator has no threshold.

## 5. Discussion

### 5.1. Advantages of using social media data for sentiment quantification

The data used in previous related studies had either low spatial resolution (or a fuzzy classification was used) or lacked quantitative information. Using questionnaires to survey residents' sentiments is costly and provides small sample sizes, making it difficult to capture residents' sentiments objectively. Inconsistent results may be obtained, and it is not possible to analyze the non-linear correlation between the built environment and sentiments. Using social media data for sentiment analysis allows for quantitative analysis of urban sentiments. Questionnaires may be subjective, with leading questions and a specific survey context. In contrast, social media data capture the residents' sentiments (90). The SKEP model enables the efficient quantification of the sentiments of urban residents. It provides a score of the sentiments and has the advantages of low cost, a large sample size, and quantitative and reproducible results (80).

### 5.2. Multilevel relationships between sentiments and the built environment

We observed differences in the relationships between sentiments and the built environment between the neighborhood and subdistrict.

At the neighborhood level, housing with a high land value and close proximity to the waterfront evoked positive sentiments, consistent with previous research (7, 8, 28). A low plot ratio and low population density resulted in negative sentiments, which is in line with residents' needs for high-quality housing. Our research also revealed the relationship between population density and sentiments. Previous research found that a high-population density worsened environmental conditions and increased noise, causing negative sentiments (30, 53). However, several studies found that an increase in population density in low-density areas improved the residents' sentiments by increasing opportunities for interpersonal interactions (54, 55). However, in compact cities like Xiamen, residents prefer low-density settlements. Reducing the population density of residential areas can improve the quality of life; thus, it is one objective of urban planners (91).

At the subdistrict level, more green spaces and commercial land improved the sentiments of the residents, whereas a large proportion of industry resulted in negative sentiments. An increase in the proportion of commercial land use significantly improved residents' sentiments, suggesting that residents enjoy the convenience offered by nearby commercial facilities (38) and are not bothered by the negative impacts, such as traffic congestion and noise, that some studies have associated with commercial land use (37). A higher proportion of the migrant population in the neighborhood significantly worsened the residents' sentiments. Previous studies have also concluded that too many foreign renters affected the living experience of local residents and made it difficult for outsiders to integrate into local life (56, 57), resulting in negative sentiments. A high road density in a subdistrict indicates a higher level of development (49), increasing traffic and meeting the travel needs of more residents.

### 5.3. Non-linear correlation between sentiments and the built environment

The GBDT model can predict complex non-linear associations and is particularly efficient when the non-linear associations differ for different independent variables (89). Figure 6 shows the relationship between different indicators and the sentiments.

The land value is a basic need of residents. These indicators had non-linear relationships with sentiments. The home owners' sentiments can be significantly affected by a change in the housing price when the price is <60% of the maximum value (about 60,000 yuan per square meter in Xiamen). At this price, most people in low-income and middle-income groups can own a house. Home owners are not affected by a change in house prices it the price is higher than 60% of the maximum value.

In contrast, the plot ratio, population density, proportion of industrial land, and the proportion of the migrant population were advanced needs and had a non-linear correlation with sentiments. The plot ratio had a threshold of 3. A plot ratio of 0–3 usually indicates low-rise and middle-rise housing. The lower the plot ratio in this range, the more spacious the living place; thus, a decrease in the plot ratio improved the residents' sentiments. When the plot ratio was more than 3 in middle-rise and high-rise residential houses, an increase in the plot ratio did not affect the housing type; therefore, there was a negligible effect on the sentiments. The lower the population density in the neighborhood, the more positive the sentiments of the inhabitants were at densities of <300 people per hectare. At greater densities, the sentiment remained relatively stable. The threshold for the proportion of industrial land within the subdistricts was 10% (e.g., industrial workers' living quarters, industrial attached neighborhoods). Above this threshold, the sentiments of the residents were not significantly affected. In contrast, below 10%, as the percentage decreased, the sentiments of the residents improved substantially, which is in line with the planning and construction criteria of keeping neighborhoods away from industrial areas to improve their quality (33, 34). When the migrant population in the neighborhood was <20%, the sentiments of the residents were largely unaffected and remained high. Between 20 and 40%, a significant increase in the migrant population caused a significant decrease in the sentiments in the neighborhood. The residents exhibited more xenophobia, and the migrant population showed a decrease in their sense of belonging (56, 57). When the proportion of the migrant population exceeded 40%, the sentiments stabilized.

In general, advanced need represents the residents' desire for a high-quality residential life and should be the focus of high-quality construction in urban renewal projects. The findings of this article indicate inequalities in the sentiments in Xiamen due to differences in the built environment of neighborhoods and subdistricts. These differences may lead to inequalities in the health of residents (92). Therefore, planners and designers should focus on meeting the various needs of urban residents to promote health equity (93). High-density development in Xiamen currently meets the needs of residents at the middle and lower levels, although residents desire a high-quality built environment with low plot ratios, low population density, low proportion of industrial land, and low proportion of the migrant population in the neighborhood and district. These factors are considered in the current people-oriented urban renewal and transformation projects in Xiamen. The non-linear correlations and thresholds obtained in this study (Table 5) can inform decision-makers in other cities to meet the needs of residents at different stages of development. This information enables the use of planning and design tools in a targeted manner to improve the living experience of residents.

## 6. Conclusion

We used social media data and the SKEP model to quantify residents' sentiments in Xiamen, China. Multilevel regression models and GBDT models were used to investigate the effects of various indicators on sentiments in the neighborhood and subdistrict and determine the non-linear correlation between different indicators and sentiments. The multilevel regression results showed that neighborhoods with a higher land value, lower plot ratio, lower population density, and water frontage were more likely to evoke positive sentiments. At the subdistrict level, more green spaces and businesses, less industry, higher building and road densities, and a smaller migrant population were more likely to result in positive sentiments. Approximately 19% of the variability in the residents' sentiments was explained by the subdistrict indicators. We used the GBDT model to derive the non-linear correlations between the sentiments and different indicators. The proportion of green space and commercial land and building and road density were linearly correlated with residents' sentiments. The land value is a basic need and exhibited a non-linear correlation with sentiments. The plot ratio, population density, the proportion of industrial land, and the proportion of the migrant population were advanced needs and had non-linear correlations with sentiments. The basic needs should be met first during planning and construction, whereas advanced needs are a direction for developing a high-quality living environment.

Quantitative analysis of sentiments can provide powerful data support for urban planners and decision-makers. This analysis is superior to using traditional questionnaires, which provide discrete data that do not reflect global attributes. Combining social media data and intelligent sentiment analysis algorithms can greatly improve the efficiency and accuracy of sentiment quantification. Priorities for planning and construction can be determined based on the needs of residents at different levels. Decision-makers should consider the correlation of different indicators with sentiments to optimize resource allocation. Future research should focus on assessing sentiments with larger sample sizes and including more built environment variables (e.g., climate, city size, etc.) to reveal the complex relationship between the urban built environment and residents' mental health from a people-centered perspective. In addition, our study on the relationship between environment and human emotions is exploratory in nature. There are currently no models that explain both multilevel and non-linear relationships. Further analyses of multilevel and non-linear relationships are needed.

## Data availability statement

The original contributions presented in the study are included in the article/supplementary material, further inquiries can be directed to the corresponding author.

## Author contributions

CF led the project and provided the idea for this research. CF and ZG designed the research and wrote the paper. CF, ZG, SL, and CJ collected, analyzed, and validated the data. ZG created the figures. LZ revised and supervised the manuscript. YC, YGu, CJ, YZ, and YGe helped with the programming. All authors contributed to the article and approved the submitted version.
